# Microarray Analysis Identifies the Potential Role of Long Non-Coding RNA in Regulating Neuroinflammation during Japanese Encephalitis Virus Infection

**DOI:** 10.3389/fimmu.2017.01237

**Published:** 2017-09-29

**Authors:** Yunchuan Li, Hao Zhang, Bibo Zhu, Usama Ashraf, Zheng Chen, Qiuping Xu, Dengyuan Zhou, Bohan Zheng, Yunfeng Song, Huanchun Chen, Jing Ye, Shengbo Cao

**Affiliations:** ^1^State Key Laboratory of Agricultural Microbiology, Huazhong Agricultural University, Wuhan, China; ^2^Laboratory of Animal Virology, College of Veterinary Medicine, Huazhong Agricultural University, Wuhan, China; ^3^The Cooperative Innovation Center for Sustainable Pig Production, Huazhong Agricultural University, Wuhan, China

**Keywords:** Japanese encephalitis virus, brain, neuroinflammation, long non-coding RNAs, microarray

## Abstract

Japanese encephalitis virus (JEV) is the leading cause of epidemic encephalitis worldwide. JEV-induced neuroinflammation is characterized by profound neuronal cells damage accompanied by activation of glial cells. Albeit long non-coding RNAs (lncRNAs) have been emerged as important regulatory RNAs with profound effects on various biological processes, it is unknown how lncRNAs regulate JEV-induced inflammation. Here, using microarray approach, we identified 618 lncRNAs and 1,007 mRNAs differentially expressed in JEV-infected mice brain. The functional annotation analysis revealed that differentially regulated transcripts were predominantly involved in various signaling pathways related to host immune and inflammatory responses. The lncRNAs with their potential to regulate JEV-induced inflammatory response were identified by constructing the lncRNA-mRNA coexpression network. Furthermore, silencing of the two selected lncRNAs (E52329 and N54010) resulted in reducing the phosphorylation of JNK and MKK4, which are known to be involved during inflammatory response. Collectively, we first demonstrated the transcriptomic landscape of lncRNAs in mice brain infected with JEV and analyzed the coexpression network of differentially regulated lncRNAs and mRNAs during JEV infection. Our results provide a better understanding of the host response to JEV infection and suggest that the identified lncRNAs may be used as potential therapeutic targets for the management of Japanese encephalitis.

## Introduction

Japanese encephalitis virus (JEV) is mosquito-borne flavivirus belonging to the family *Flaviviridae*, which includes several other important human pathogens such as Zika virus, dengue virus, West Nile virus, yellow fever virus, and Murray Valley encephalitis virus (1). JEV is the most prevalent cause of viral encephalitis in most countries of South and East Asia and the Western Pacific (2). Approximately 35,000–50,000 cases of Japanese encephalitis are reported annually in the region with 10,000 deaths, and nearly half of the survivors suffer from perpetual neuropsychiatric sequelae (3, 4).

During infection, JEV invades the central nervous system, wherein virus replication triggers a massive inflammatory response, and subsequently causes neuronal cell death (5). The crucial factor in JEV-induced neuroinflammation is the uncontrolled activation of microglia, which release proinflammatory cytokines and chemokines such as interleukin (IL)-1β, IL-6, monocyte chemotactic protein 1, tumor necrosis factor alpha (TNF-α), and chemokine (C-C motif) ligand 5 (6, 7). The higher levels of proinflammatory mediators appear to commence an irreversible inflammatory response leading to neuronal death (4). Many studies have shown that microglia can be directly infected with JEV, and serves as long-lasting reservoir for JEV (8). Therefore, the activation of microglia is a key event during JEV-caused neuroinflammation. Previously, we have investigated various molecular mechanisms of JEV pathogenesis (9–12); however, further studies are still required to advance the understanding of JEV-induced neuroinflammation.

Recent advances have revealed that both mouse and human genomes contain a large number of long non-coding RNA (lncRNA) genes (13). lncRNAs have been implicated as regulators of diverse cellular processes such as regulation of cell cycle, chromatin structure, and RNA stability (14–16). However, the precise molecular mechanisms by which lncRNAs act are largely unknown. Recently, the transcriptomic analysis has revealed that a large number of lncRNAs were differentially expressed upon infection with severe acute respiratory syndrome coronavirus and enterovirus 71 (17, 18). Furthermore, several lncRNAs have been reported to be involved in virus replication. For instance, an lncRNA, NRAV, can regulate the replication of influenza A virus through inhibition of interferon-stimulating genes such as IFITM3 and MxA (19). Many lncRNAs have also been shown to modulate inflammatory response (20–23). Of these, Lethe, a pseudogene lncRNA, interacts with nuclear factor-kappa B to inhibit the binding of RelA with DNA and to suppress the activation of downstream signaling cascades (23). These findings suggest that lncRNAs play important roles in virus infection and inflammatory response. Thus, further efforts toward accurate annotation and functional significance of lncRNAs will be critical for our understanding of disease processes.

Among non-coding RNAs, microRNAs (miRNAs) have been extensively studied in posttranscriptional regulation of gene expressions in various biological processes (24). Accumulating evidence also suggests a key role for miRNAs in various neuroinflammatory diseases (25, 26), including Japanese encephalitis (9). However, the role of lncRNAs in JEV-induced neuroinflammation and virus replication is still unknown.

In the present study, transcriptomic profiling of lncRNAs and mRNAs from JEV-infected mice brain was performed using a microarray platform. Our results identified JEV-induced changes in expression patterns of lncRNAs and mRNAs in JEV-infected and non-infected mice brain samples. This study may provide new insights into the pathogenic mechanisms associated with JEV infection.

## Materials and Methods

### Virus Isolation and Titration

Postnatal 3–4-day-old suckling BALB/C mice were infected with P3 strain of JEV. Upon onset of clinical manifestation of JEV such as poor brain response, limb paralysis, and whole body tremor, mice were killed and their brains were excised. Brain homogenate prepared in Dulbecco’s modified Eagle’s medium (DMEM) was centrifuged at 10,000*g* to remove cellular debris. The resultant suspension was filtered through 0.22 µm sterile filters to obtain viral suspension. Immediately, aliquots of filtered virus suspension were stored at −80°C until further use. The virus titer was determined by plaque formation assay on baby hamster Syrian kidney (BHK-21) cells as described previously (10).

### JEV Infection

Adult BALB/C mice (6 weeks old) were purchased from Hubei Provincial Center for Disease Control and Prevention, Wuhan, China. Mice were randomly assigned to two groups (*n* = 3 for each group): the JEV-infected group and the control group. For the infection group, mice were inoculated intracranially with 200 plaque-forming units (PFU) JEV (P3 strain) in 20 µl DMEM, whereas mice belonging to the control group were injected intracranially with equal volume of DMEM. On day 5 postinfection, mice infected with JEV showed signs of acute encephalitis. Mice from both groups were euthanized, and brain samples were collected for further studies. All animal experiments were performed following the National Institute of Health Guide for the Care and Use of Laboratory Animals, and the experimental protocols were approved by the Research Ethics Committee of College of Veterinary Medicine, Huazhong Agricultural University, Hubei, Wuhan, China (No. S02914040M). All of our virus experiments are under Biosafety Level 2 containment. We performed double-blind procedure in our *in vivo* experiments, blinding investigators, participants, and outcome assessors.

### Cell Culture

The RAW264.7 cells were purchased from the American Type Culture Collection, and the BV2 cells were kindly provided by Professor Yuanan Lu from University of Hawaii, Manoa, USA (27). The cells passage numbers were below 20. BV2 and RAW264.7 cells were cultured and maintained in DMEM that was supplemented with 10% (v/v) heat-inactivated fetal bovine serum, 100 U/ml penicillin, and 100 mg/ml streptomycin sulfate at 37°C in 5% CO_2_ atmosphere. Cells were seeded in multiwell plates, and grown to 80% confluency. Non-adherent cells were removed by washing with non-supplemented DMEM prior to further treatment. All of our virus experiments are under Biosafety Level 2 containment.

### Immunofluorescence Analysis

The cells were mock-infected or infected with JEV P3 strain at multiplicity of infection (MOI) of 5 for 2 h. At 12, 24, and 36 h postinfection, cells were fixed and blocked with 10% bovine serum albumin in phosphate-buffered saline (PBS, pH 7.2) for 30 min. Then, cells were incubated with monoclonal antibody recognizing JEV NS5 (5 ng/ml for immunofluorescence assay, prepared by our laboratory) for 1 h. After washing three times with PBS, cells were incubated with Alexa Fluor 488-conjugated secondary antibody (Invitrogen) for 30 min. Cells nuclei were stained with 4′,6-diamidino-2-phenylindole dihydrochloride (Invitrogen). The staining was observed using a fluorescence microscope (Zeiss) with 20× magnification.

### RNA Interference

Small-interfering RNAs (siRNAs) corresponding to the sequence of lncRNA-E52329 and lncRNA-N54010, which were used to inhibit endogenous expression of lncRNA-E52329 and lncRNA-N54010, and the negative control siRNAs, which exhibited no downregulation of any mouse genes, were synthesized by Gene pharma. Transfection was performed with Lipofectamine 2000 (Invitrogen). Cells were transfected with 50 nM of each siRNA. The sequence of all siRNAs used in this study is listed in Table S6 in Supplementary Material.

### RNA Extraction and Quantitative Real-time PCR

Total RNA was extracted from mouse brain or treated BV2 or RAW264.7 cells using TRIzol reagent (Invitrogen), and subsequently, was reverse transcribed into cDNA using First Strand cDNA Synthesis Kit (TOYOBO) following the manufacturer’s instructions. Quantitative real-time PCR was performed using a ViiA™ 7 Real-time PCR System (Applied Biosystems) and SYBR Green Real-time PCR Master Mix (TOYOBO). Amplification was performed for 2 min at 50°C and 10 min at 95°C, followed by 40 cycles of 95°C for 15 s, 60°C for 15 s, and 72°C for 30 s. The relative expression levels of lncRNAs, TNF-α, IL-6, IL-1β, and IFN-β were normalized to that of β-actin within each sample using the 2^−ΔΔCt^ method. The specific primers for lncRNAs, TNF-α, IL-6, IL-1β, IFN-β, and β-actin are listed in Table S6 in Supplementary Material.

### Microarray Analysis

Total RNA was extracted and purified using miRNeasy Mini Kit and RNase-Free Dnase Set. Biotinylated cDNA was prepared according to the standard Affymetrix protocol from 250 ng total RNA by using GeneChip^®^ WT PLUS Reagent Kit. The resultant cDNA was then transcribed to generate cRNA, which is reverse transcripted to yield second-cycle cDNA, and then fragmented. Following labeling, 5.5 μg of cDNA was hybridized for 16 h at 45°C on GeneChip Mouse EG 1.0 ST Array in GeneChip^®^ Hybridization Oven 645. GeneChips were washed and stained in the Affymetrix Fluidics Station 450. The array was scanned by GeneChip2 Scanner 3000 7G. The probe array was scanned by Affymetrix^®^ GeneChip Command Console. The gene expression levels were normalized by Expression Console. Expression data were generated by Affymetrix Expression Console software and normalized by the Robust Multi-chip Average (RMA) method. RVM *t*-test performed by BRB-Array Tools (v4.3.2) was applied to filter the differentially expressed genes for the control and experimental group. After significant analysis and false discovery rate (FDR) analysis, we selected the differentially expressed genes according to the *P*-value and fold change threshold (absolute ratio). *P*-value <0.05 and fold change >2 for mRNA or fold change >3 for lncRNA, were considered as significant difference. The lncRNAs and mRNAs were annotated through the Affymetrix Power Tools from the database of Refseq, Ensembl, and Non-code. The microarray data are available on NCBI GEO database, and GEO accession number is GSE94789.

### Heat Map and Functional Analysis of Differentially Expressed Genes

The statistical heat map of the two groups was constructed using software Cluster (Version 3.0) to show the difference of gene fold changes between both groups.

To gain insight into the functions of differentially expressed genes, associated gene ontology (GO) terms were identified using DAVID Bioinformatics Resources (version 6.7, http://david.abcc.ncifcrf.gov/). It can organize genes into hierarchical categories and uncovers the gene regulatory networks on the basis of biological process and molecular function (28, 29). Specifically, we used two-side Fisher’s exact test and χ^2^ test to classify the GO category, and the FDR (30) was calculated to correct the *P*-value. Smaller the value of the FDR, smaller will be the error in judging the *P*-value. We computed *P*-values for the GOs of all differentially regulated genes. Enrichment provides a measure of significance of the function: as the enrichment increases, the corresponding function is more specific, which helps us to find those GOs with more concrete function description in the experiment.

We also used DAVID software to analyze the signaling pathways of differentially expressed genes according to KEGG, Biocarta, and Reatome. The significance was determined using the Fisher’s exact test and χ^2^ test, and the threshold of significance was defined by *P*-value and FDR (31–33).

### Coexpression Network

The coexpression networks were built according to normalized signal intensity of specific gene expressions. For each pair of genes, the Pearson correlation was calculated, and the significant correlation pairs were chosen to construct the network (34).

The gene coexpression network was constructed in experimental group and control group, respectively. Within the network analysis, a degree is the simplest and most important measure of centrality of a gene within a network and determines the relative importance. A degree is defined as the number of directly linked neighbors.

Degree in experimental group was recorded as exp_degree, whereas in control group was recorded as con_degree. A clustering coefficient is a measure of the degree to which nodes in a graph tend to cluster together. It was calculated by the local measure (35). To exclude other genes’ impact in each coexpression network, we further performed normalization of the degree, i.e., divided by maximum value of the gene degree in each network [Normalized degree(i) = Degree(i)/Degree(Max)]. Then, the difference value of a gene’s normalized degree (delta normalized degree, represented as |diffK|) was calculated between the two coexpression networks.

While considering different networks, core regulatory factors were determined based on degree differences between the two class samples (36). Considering our pathway analyses, we chose some genes of our interest, and functions of most of the selected genes were related to inflammation. The cytoscape software (version 5.3) was used to build the interaction map.

### Pull-Down Assay with Biotinylation RNA

The lncRNA E52329 and N54010 were amplified by polymerase chain reaction. The lncRNAs were transcribed (T7 MEGAscript™, Ambion) and biotinylated (Pierce™ RNA 3′ End Desthiobiotinylation, Thermo Scientific) according to the manufacturer’s protocol. The JEV-infected BV2 cells were lysed in radioimmunoprecipitation assay buffer (Sigma) containing protease and phosphatase inhibitors (Roche). For pull-down experiments, biotin-labeled RNA or antisense-RNA were incubated with streptavidin magnetic beads on a rotator at room temperature for 30 min in the cellular extract solutions, then washed with PBS for five times, and the resultant pellet was extracted RNA for qRT-PCR (37).

### Western Blotting

Total mice brain tissues lysates were generated using radioimmunoprecipitation assay buffer (Sigma) containing protease and phosphatase inhibitors (Roche). Protein concentrations were measured with a BCA Protein Assay Kit (Thermo Scientific). Equal protein quantities were separated by SDS-PAGE and transferred to a polyvinylidene fluoride membrane (Millipore) using a Mini Trans-Blot Cell (Bio-Rad). Blots were probed with the relevant antibodies, and proteins were detected using the ECL reagent (Thermo Scientific). Mouse monoclonal antibodies against JEV NS5 were generated in our laboratory. Commercially obtained antibodies used were: mouse monoclonal antibody against GAPDH, rabbit polyclonal antibodies against β-tubulin, MKK4, IκBα, phosphor-MKK4-Thr261 (ABclonal Technology), phosphor-IκBα-Ser32, phosphor-SAPK/JNK-Thr183/Tyr185 (CST technology), and horseradish peroxidase-conjugated antimouse secondary antibodies (Boster).

### Plaque Assay

BV2 and RAW264.7 cells were transfected with siE52329, siN54010, or their controls (final concentration, 50 nM) for 24 h, and subsequently infected with JEV at MOI of 5. At 12, 24, and 36 h postinfection, cell supernatants were harvested, serially diluted, and then used to inoculate monolayers of BHK-21 cells. After removal of unbound JEV virus particles, BHK-21 cells were further incubated for 3–5 days, and plaques identified. The visible plaques were counted and viral titers calculated. All data are expressed as the mean of triplicate samples. All of our virus experiments are under Biosafety Level 2 containment.

### ELISA

The culture supernatants were collected from treated cells at the indicated time points and stored at −80°C. The protein levels of TNF-α, IL-1β, and IL-6 in cell cultures or mouse brain tissue lysates were measured by ELISA kits (eBioscience) following the manufacturer’s instructions.

### Hematoxylin–Eosin (H&E) Staining, Immunohistochemistry (IHC), and the Terminal Deoxynucleotidyl Transferased UTP Nick End Labeling (TUNEL) Assay

Mice were anesthetized with ketamine-xylazine (0.1 ml/10 g of body weight) and perfused with PBS, followed by 4% paraformaldehyde. Brain tissues were removed and embedded in paraffin for coronal sections. The sections were used for H&E staining, IHC, and the TUNEL assays as described previously (9). For IHC experiment, tissue sections were incubated overnight at 4°C with primary antibodies against ionized calcium-binding adapter molecule 1 (IBA-1) (Wako), glial fibrillary acidic protein (GFAP) (Dako), and neuron-specific nuclear protein NeuN (Chemi-Con) at concentrations indicated in the manufacturer’s guidelines. After washing, slides were incubated with antimouse horseradish peroxidase-conjugated secondary antibodies, washed, and 3,3′-diaminobenzidine (Vector Laboratories) was used for color development. For TUNEL assay, an *In Situ* Cell Death Detection Kit (Roche) was used according to the manufacturer’s instructions.

### Statistical Analysis

All results are expressed as mean ± SEM. Statistical analysis was performed by GraphPad Prism 5 (GraphPad Software, San Diego, CA, USA). Differences were analyzed for statistical significance using two-sided unpaired t test for two groups or multiple comparison one-way of variance (ANOVA) for more than two groups (Bonferroni’s multiple comparison test, *P* < 0.05).

## Results

### Validation of Successful JEV Infection in Mice Brain

**Figure 1:**
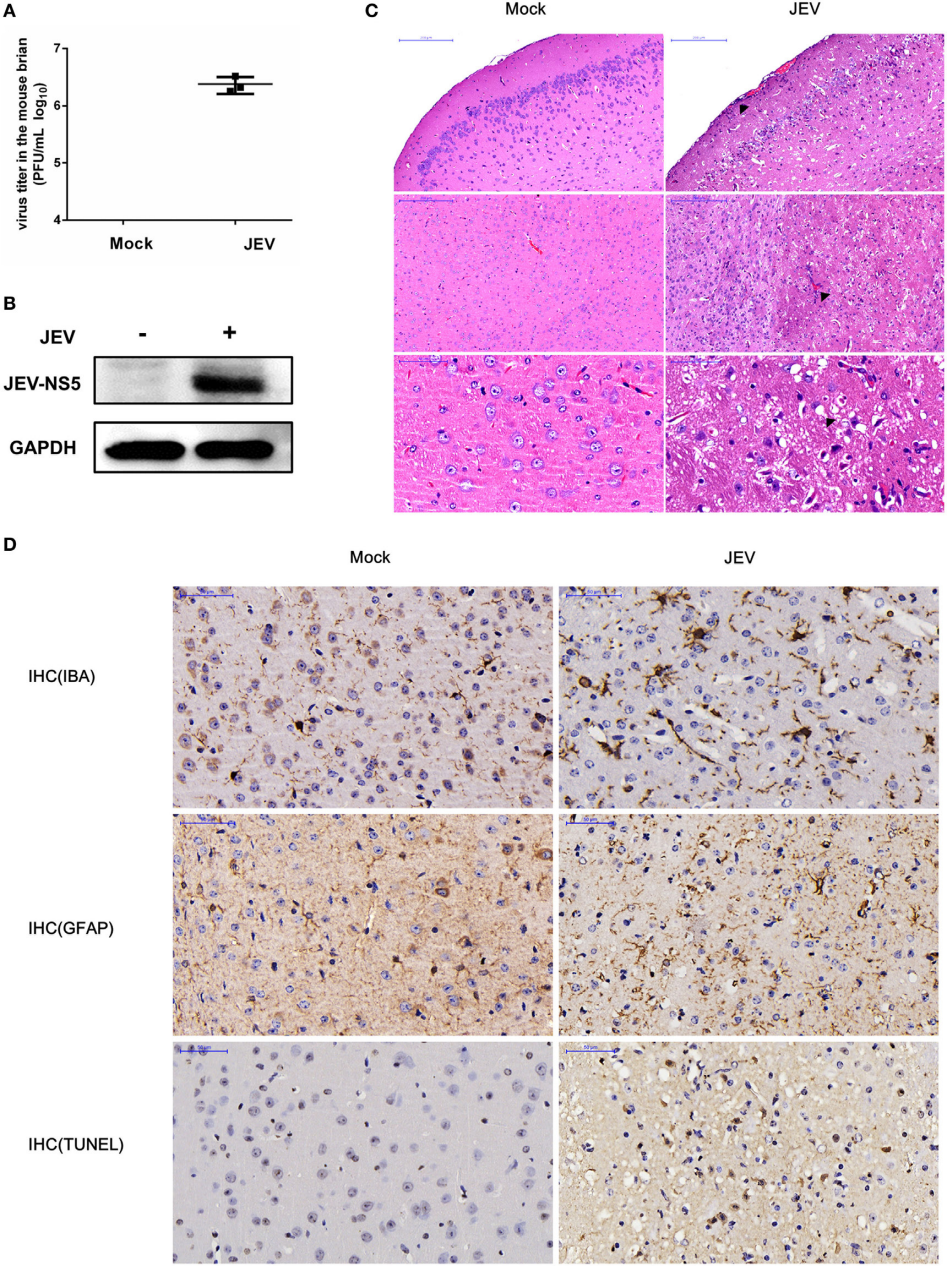
Validation of successful Japanese encephalitis virus (JEV) infection in mice brain. For the infection group, mice were inoculated intracranially with 200 PFU JEV in 20 µl Dulbecco’s modified Eagle’s medium (DMEM), whereas mice belonging to the control group were injected intracranially with equal volume of DMEM. On day 5 postinfection, mice from both groups were euthanized, and brain samples were collected. **(A)** Quantification of infectious virus titer in mice brain by plaque assay. **(B)** Expression analysis of JEV NS5 by Western blot. **(C)** Hematoxylin–eosin (H&E) staining of brain sections to examine the histopathological changes. Scale bar = 200 µm. **(D)** Immunohistochemistry (IHC) analysis of brain sections to determine the activation of microglia and astrocytes using anti-ionized calcium-binding adapter molecule (anti-IBA) and antiglial fibrillary acidic protein (anti-GFAP) antibodies, respectively. Cell death was examined by terminal deoxynucleotidyl transferased UTP nick end labeling (TUNEL) assay. Scale bar = 50 μm. Integrated option density (IOD) analysis was performed to quantify the results of staining. Data represent the ranges observed from three sections from three mice in each group.

To start our analysis, we first established a mouse model for JEV infection and then collected samples for the microarray analysis (Figure S1 in Supplementary Material). To confirm whether the mice have successfully acquired the JEV infection, we examined the virus replication in mice brain samples using plaque assay and Western blot analysis. The results showed that the infected mice had a high virus titer in the brain tissues (Figure 1A), and these tissues exhibited an increased expression of JEV NS5 (Figure 1B). To visualize the effect of JEV infection in mice brain, we examined the brain tissue sections processed for H&E staining and IHC analysis. The data demonstrated that JEV-infected mice presented the emblematic histopathological features of encephalitis, whereas no pathological changes were noticed in the mice belonging to control group (Figure 1C). Furthermore, an aberrant increase in number of astrocytes and microglia was detected by IHC using anti-GFAP and -IBA-1 antibody, respectively (Figure 1D). Taken together, these findings indicate the establishment of a productive JEV infection in the mice brain.

### Overview of lncRNA and mRNA Microarray Data

**Figure 2:**
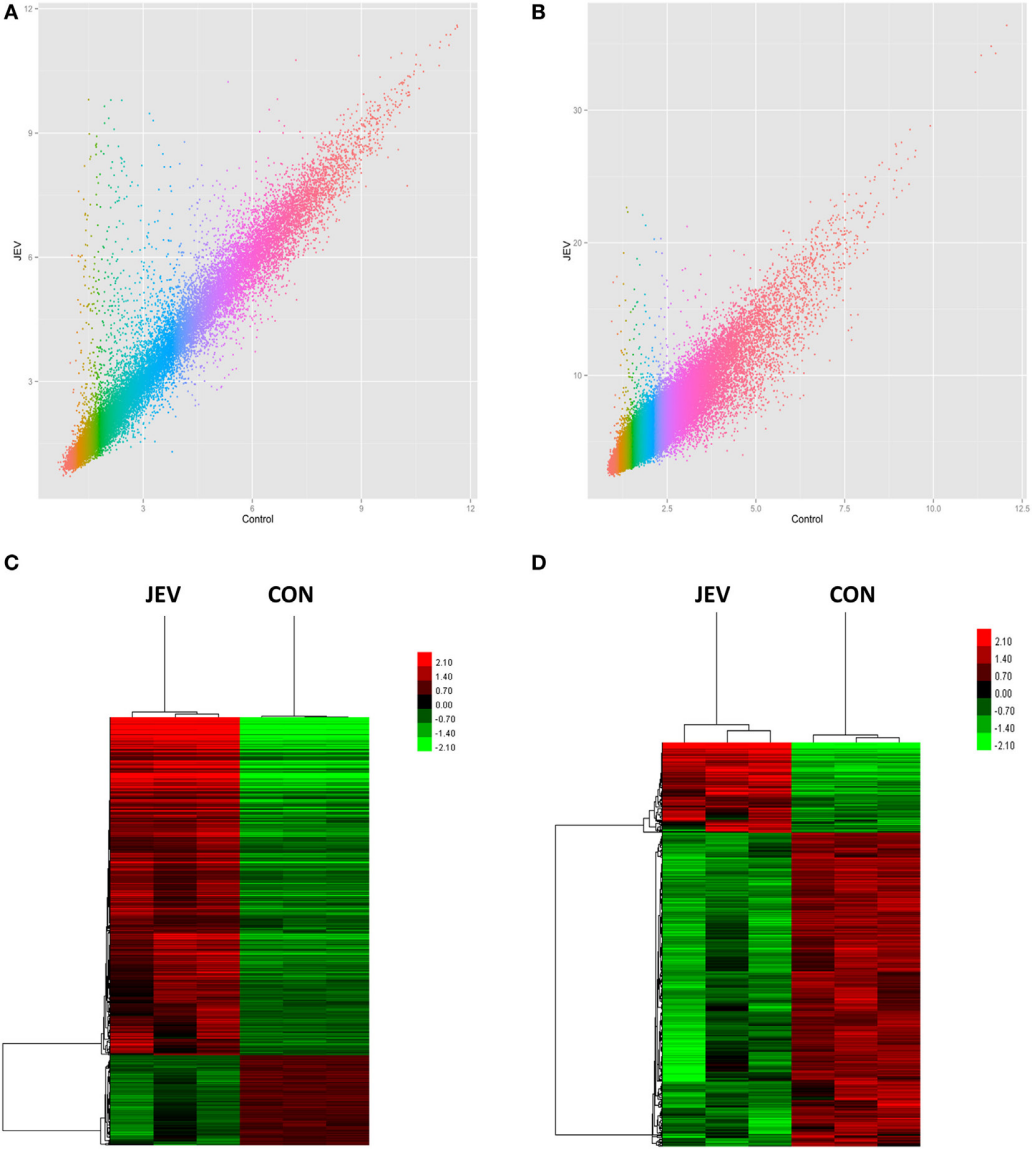
Expression profiles of mRNAs and long non-coding RNAs (lncRNAs) in Japanese encephalitis virus (JEV)-infected and mock-infected mouse brain. **(A,B)** Scatter plot to show the variation in mRNA **(A)** and lncRNA **(B)** expressions. The values of X and Y axes are the mean normalized signal values in each group (log 2-scaled). **(C,D)** Hierarchical clustering of differently expressed mRNAs **(C)** and lncRNAs **(D)** between the two groups. Red color indicates relatively higher expression, whereas green color indicates relatively lower expression.

To identify changes in the expression levels of lncRNAs and mRNAs in mice brain infected with JEV, total RNA was extracted from JEV-infected or mock-infected mice brain. The array which covers 30,772 lncRNAs and 16,417 protein-coding transcripts from data sources such as Ensembl, fRNAdb, lncRNAdb, NOCODE4.0, and UCSC known gene, was used to detect lncRNAs and mRNAs in the mice brain samples (Table S1 in Supplementary Material). Scatter plot was used to show variation in lncRNAs and mRNAs expressions between JEV-infected and mock-infected mice brain samples (Figures 2A,B). RVM *t*-test revealed that 518 lncRNAs and 1,007 mRNAs were differentially expressed in a significant manner between the two groups. Among the differentially expressed lncRNAs and mRNAs, 116 lncRNAs and 795 mRNAs were found to be upregulated, whereas 402 lncRNAs and 212 mRNAs exhibited a downregulated pattern (*P* < 0.05; fold change >2 for mRNA or fold change >3 for lncRNA, Table 1; Table S2 in Supplementary Material). The hierarchical clustering analysis was also employed to show the differential expressions of lncRNAs and mRNAs between both groups of samples (Figures 2C,D).

**Table 1:** Statistical analysis of differentially regulated mRNAs and lncRNAs.

	Upregulated	Downregulated	Total
mRNA	795	212	1,007
lncRNA	116	402	518

### GO Terms and Pathways Analyses

To better understand the functions of differentially regulated mRNAs and lncRNAs in JEV-induced inflammation, GO term analysis of these mRNAs and lncRNAs was performed. We found that highly enriched GO categories for mRNAs included, but were not confined to, phosphoproteins, nuclear functions, signal transduction, acetylation, and nucleotide binding (Figure S2A in Supplementary Material). The most significant GOs associated with upregulated lncRNAs were related to innate immune response, apoptotic process, and inflammatory response (Figure S2C in Supplementary Material). The top 10 significantly upregulated and downregulated GO terms linked with differentially regulated mRNAs and lncRNAs are presented in Figures S2A–D and Tables S3 and S4 in Supplementary Material.

Pathways analysis of differentially expressed 1,007 mRNAs and 518 lncRNAs was performed using the KEGG (Kyoto Encyclopedia of Genes and Genomes). Several important signaling pathways, i.e., those of Toll-like receptor, Jak-STAT, MAPK, chemokines, Herpes simplex and influenza A infection, and PI3K-Akt, were markedly activated by differentially expressed mRNAs and lncRNAs (Figures S2A,C in Supplementary Material). The top 10 upregulated and downregulated pathways associated with these mRNAs and lncRNAs are shown in Figures S2A–D and Tables S3 and S4 in Supplementary Material.

### Coexpression Network Analysis

To investigate the correlation of lncRNAs and mRNAs, we constructed the lncRNA-mRNA coexpression network in each of the two separate groups (Table S5 in Supplementary Material). We used biostatistics to calculate the possibility of interaction between these genes. The solid line between the two nodes strands for positive correlation, whereas the dotted line indicates negative correlation (Figure S3 in Supplementary Material). The clustering coefficient represents the density of the gene in the network. Higher value of the clustering coefficient indicates more dependent interaction with other genes in the network or vice versa. The genes with higher degree and higher clustering coefficient in both groups are listed in Table 2.

**Table 2:** Important differentially expressed lncRNAs in JEV-infected group.

lncRNA_id	Database	Clustering coefficient	Degree	Style	Type
ENSMUST00000124479	Ensembl	0.71428571	8	Up	Non-coding
ENSMUST00000125015	Ensembl	0.83870968	32	Up	Non-coding
ENSMUST00000128411	Ensembl	0.64333333	25	Up	Non-coding
ENSMUST00000129916	Ensembl	0.65873016	28	Up	Non-coding
ENSMUST00000138093	Ensembl	0.86666667	15	Down	Non-coding
ENSMUST00000139110	Ensembl	0.65763547	29	Up	Non-coding
ENSMUST00000139982	Ensembl	0.77619048	21	Up	Non-coding
ENSMUST00000152329	Ensembl	0.70760234	19	Up	Non-coding
ENSMUST00000155744	Ensembl	0.78571429	29	Up	Non-coding
ENSMUST00000160521	Ensembl	0.69590643	19	Up	Non-coding
NONMMUT000396	NOCODE4.0	0.68181818	33	Down	Non-coding
NONMMUT003879	NOCODE4.0	0.68571429	15	Up	Non-coding
NONMMUT009926	NOCODE4.0	0.80769231	13	Up	Non-coding
NONMMUT014378	NOCODE4.0	0.70289855	24	Down	Non-coding
NONMMUT019757	NOCODE4.0	0.70344828	30	Up	Non-coding
NONMMUT026104	NOCODE4.0	0.76811594	24	Down	Non-coding
NONMMUT027272	NOCODE4.0	0.86666667	15	Down	Non-coding
NONMMUT032524	NOCODE4.0	0.82051282	27	Up	Non-coding
NONMMUT033958	NOCODE4.0	0.82105263	20	Up	Non-coding
NONMMUT035523	NOCODE4.0	0.73333333	10	Down	Non-coding
NONMMUT035543	NOCODE4.0	0.82153846	26	Down	Non-coding
NONMMUT035543	NOCODE4.0	0.82153846	26	Down	Non-coding
NONMMUT047777	NOCODE4.0	0.58181818	11	Down	Non-coding
NONMMUT052217	NOCODE4.0	1	12	Down	Non-coding
NONMMUT054010	NOCODE4.0	0.66666667	7	Up	Non-coding
NONMMUT055554	NOCODE4.0	0.68735632	30	Up	Non-coding
NONMMUT057145	NOCODE4.0	0.74074074	27	Up	Non-coding
NONMMUT057146	NOCODE4.0	0.67753623	24	Up	Non-coding
NONMMUT060535	NOCODE4.0	0.75666667	25	Down	Non-coding
NONMMUT057235	NOCODE4.0	0.70952381	21	Down	Non-coding
NONMMUT061412	NOCODE4.0	0.83870968	32	Down	Non-coding

**Figure 3:**
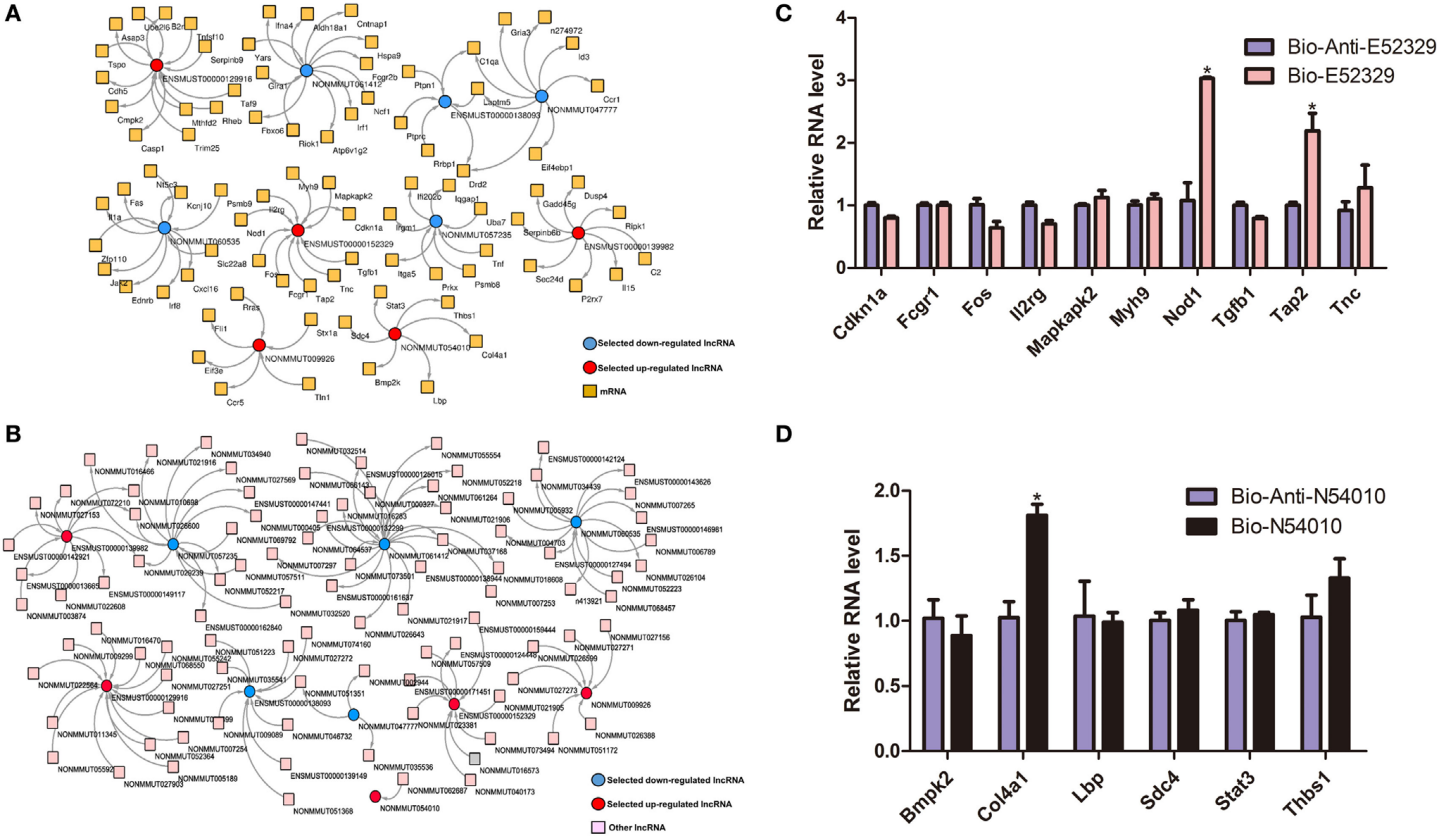
The coexpression network of significantly regulated long non-coding RNAs (lncRNAs) and mRNAs. **(A)** lncRNA-mRNA network. **(B)** lncRNA-lncRNA network. Red color specifies selected upregulated lncRNAs, and blue color indicates downregualted lncRNAs. Yellow color denotes mRNAs, whereas pink designates other lncRNAs. **(C)** Detection of indicated mRNAs using quantitative real-time PCR in the sample pulled down by biotin-labeled E52329. **(D)** Detection of indicated mRNAs using quantitative real-time PCR in the sample pulled down by biotin-labeled N54010.

In order to interrogate interactions between the genes related to inflammation, we selected some of the lncRNA genes that may participate in the inflammatory pathways, and then built the lncRNA-mRNA and lncRNA-lncRNA coexpression networks (Figures 3A,B). To confirm our coexpression network analysis, we chose lncRNA E52329 and N54010 and performed RNA pull down experiments. Our results showed that Nod1, Tap2, and Col4a1 were pulled down by indicated bio-lncRNAs compared with bio-anti-lncRNAs (Figures 3C,D). In order to determine that which pathway plays an important role in our lncRNA network, we used pathway studio (version 10) and KEGG pathway to predict the possibility of pathway enrichment. The findings showed that MKK4/7 is the most enriched pathway.

An altered gene status in the coexpression network between JEV-infected and mock-infected groups suggest that the gene may perform a very important function during the JEV infection. Therefore, we compared the status of genes in both groups, and the genes exhibiting the most significant difference are enlisted in Table 2.

### Validation of Differentially Regulated lncRNAs by Quantitative Real-time PCR

**Figure 4:**
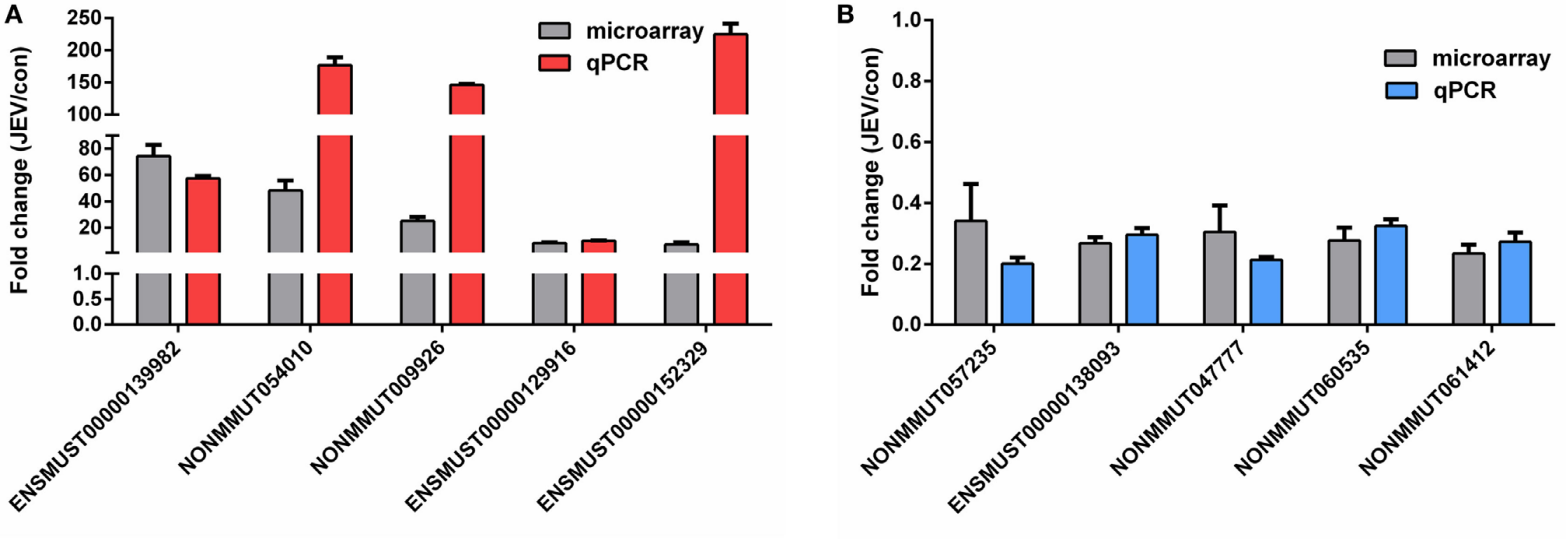
Validation of the microarray data using quantitative real-time PCR. **(A,B)** Mice were infected with Japanese encephalitis virus (JEV) or mock infected with Dulbecco’s modified Eagle’s medium (DMEM), and brain samples were collected at 5 days postinfection for analysis of selected long non-coding RNAs (lncRNAs). The levels of upregulated lncRNAs **(A)** and downregulated lncRNAs **(B)** were detected by quantitative real-time PCR. All data are representative of three independent experiments.

The differential expression levels of lncRNAs found in our microarray analysis were validated by quantitative real-time PCR. From the list of differentially regulated lncRNAs, we randomly selected five upregulated and five downregulated lncRNAs for their expression validation in mice brain tissues. Our results obtained from quantitative real-time PCR were analogous to those observed in microarray data (Figures 4A,B). We also detected the expression levels of lncRNAs in JEV-infected BV2 cells. Most of the lncRNAs results showed the same tendency as were observed in mice brain samples after JEV infection (data not shown). However, some of the lncRNAs were not detected in BV2 cells, which may happen due to tissue or cell specificity (38).

### Regulation of JEV-Mediated Inflammatory Cytokine Production and Influence on Virus Titer by lncRNAs

**Figure 5:**
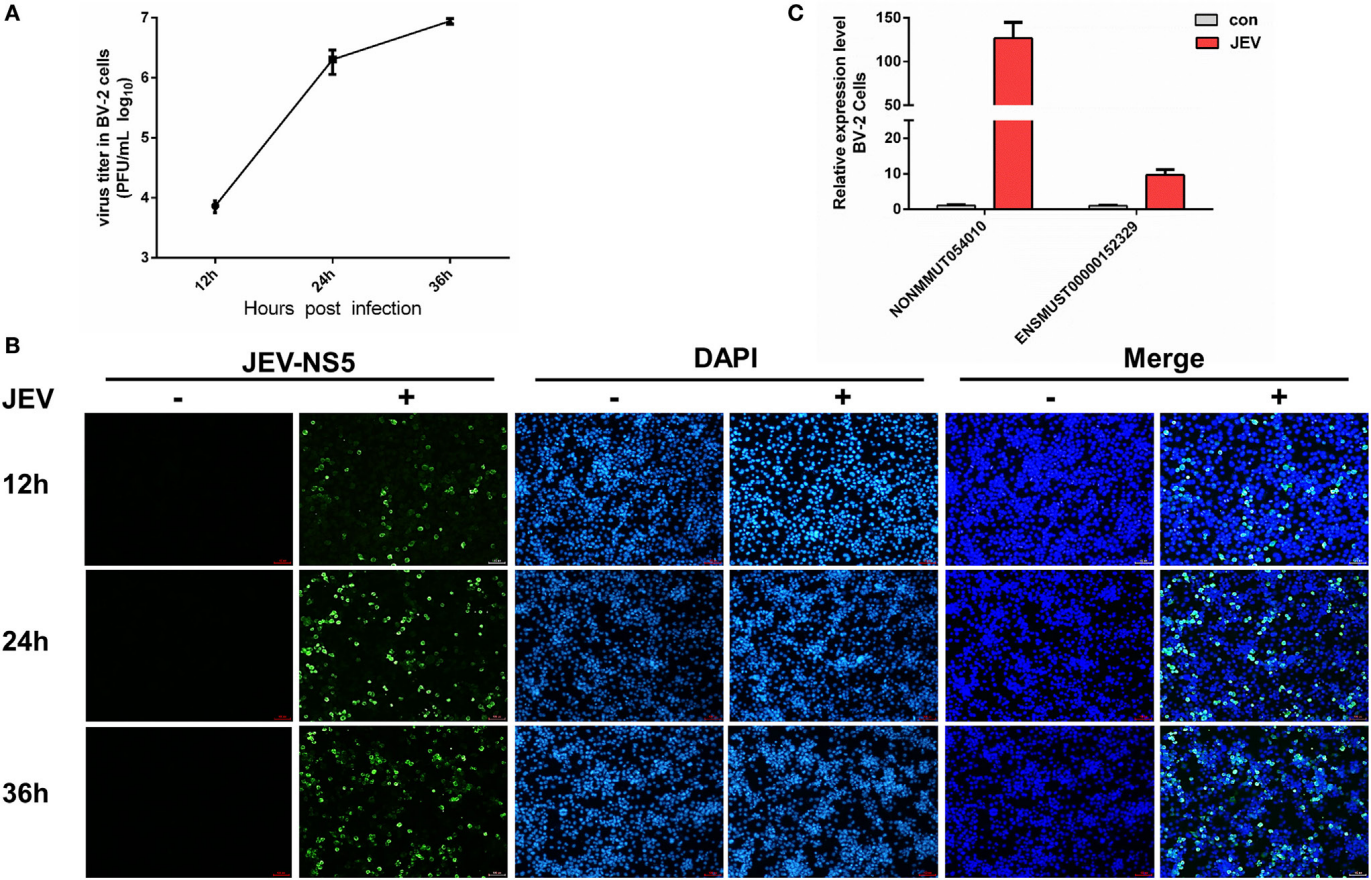
Detection of selected long non-coding RNAs (lncRNAs) in BV2 cells. **(A)** BV2 cells were infected with Japanese encephalitis virus (JEV) at multiplicity of infection (MOI) of 5 for the indicated times, and titers of infectious virus in the culture supernatants were determined by plaque assay. **(B)** BV2 cells were infected with JEV at MOI of 5 for 2 h. At 12, 24, and 36 h postinfection, cells were fixed and subjected to indirect immunofluorescence to detect JEV NS5 protein (green). Nuclei are shown by 6-diamidino-2-phenylindole dihydrochloride (DAPI, blue) staining. The images of the cells were acquired with a fluorescence microscope (Zeiss) with 20× magnification. Scale bar = 50 µm. **(C)** BV2 cells were infected with JEV at MOI of 5 for 2 h. The expression levels of lncRNAs NONMMUT054010 and ENSMUST00000152329 were detected by quantitative real-time PCR. All data are representative of three independent experiments.

**Figure 6:**
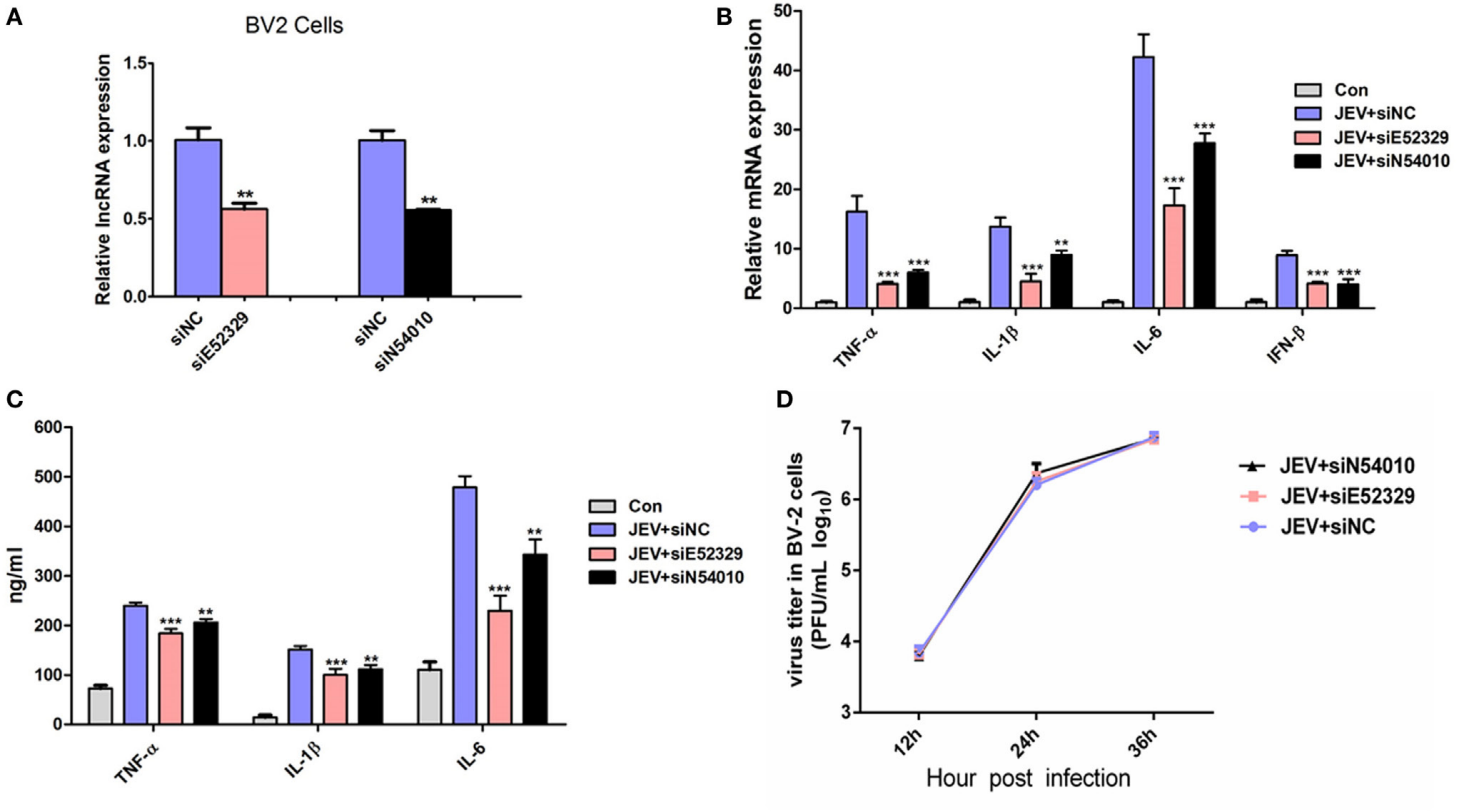
Selected long non-coding RNAs (lncRNAs) regulate Japanese encephalitis virus (JEV)-mediated production of inflammatory cytokines. **(A)** BV2 cells were transfected with siE52329, siN54010, or their non-specific control small-interfering RNA (siRNA, final concentration, 50 nM) for 24 h, and then expression levels of lncRNA NONMMUT054010 and ENSMUST00000152329 were detected by quantitative real-time PCR. **(B,C)** BV2 cells were transfected with siE52329, siN54010, or their non-specific control siRNA (final concentration, 50 nM) for 24 h, and then infected with JEV at multiplicity of infection (MOI) of 5 for 24 h. The mRNA **(B)** and protein **(C)** levels of tumor necrosis factor alpha (TNF-α), interleukin (IL)-6, and IL-1β were analyzed by quantitative real-time PCR and ELISA, respectively. IFN-β mRNA level was determined by quantitative real-time PCR. **(D)** BV2 cells were transfected with siE52329, siN54010, or their non-specific control siRNA (final concentration, 50 nM) for 24 h, and then infected with JEV at MOI of 5 for the indicated times. The titers of infectious virus in the culture supernatants were determined by plaque assay. Data were expressed as means ± SEM from three independent experiments. One-way ANOVA with subsequent Bonferroni’s multiple comparison. All data are representative of three independent experiments.

To discern a possible role of differentially regulated lncRNAs in JEV-induced inflammation, we selected two lncRNAs (lncRNA E52329, lncRNA N54010) form the data validated using mice brain samples. Since JEV infection is associated with marked activation of glial cells and exorbitant release of inflammatory cytokines (39), we chose mouse microglial cells (BV2 cells) for further analysis. To determine whether BV2 cells were permissive to JEV, the successful infection of BV2 cells was verified by plaque assay (Figure 5A) and immunofluorescence analysis (Figure 5B). Next, we examined the expression levels of selected lncRNAs in JEV-infected BV2 cells by quantitative real-time PCR. Our results were concordant to those as observed in JEV-infected mice brain samples (Figure 5C). To examine whether lncRNA E52329 and N54010 are involved in JEV-mediated inflammatory process, the effect of these lncRNAs on the regulation of inflammatory cytokine production was determined. We analyzed the outcomes of silencing of these two lncRNAs using lncRNA-specific siRNAs in BV2 cells. First, we confirmed that the siRNAs significantly inhibited the expressions of lncRNAs in BV2 cells (Figure 6A). To determine the role of lncRNAs in inflammatory cytokine production, the cells were transfected with siE52329, siN54010 or non-specific control siRNA, and then infected with JEV. The results revealed that knockdown of these lncRNAs significantly decreased the production of inflammatory cytokines at mRNA and protein levels (Figures 6B,C). Furthermore, we found that treatment of cells with siE52329 or siN54010 did not exhibit any effect on virus replication in JEV-infected BV2 cells, as viral titers were similar to those in control cells (Figure 6D). Furthermore, to examine whether these two lncRNAs are microglia specific, we selected another immune cell line (RAW264.7) as a control. The expression pattern of lncRNAs and the effects of lncRNAs-specific siRNAs on inflammatory cytokine production and virus replication were analogous to those observed in BV2 cells (Figures S4A–D in Supplementary Material). Thus, these data indicate that lncRNAs participates in regulating JEV-mediated inflammation.

### Validation of MKK4 and JNK Expressions by Western Blotting

**Figure 7:**
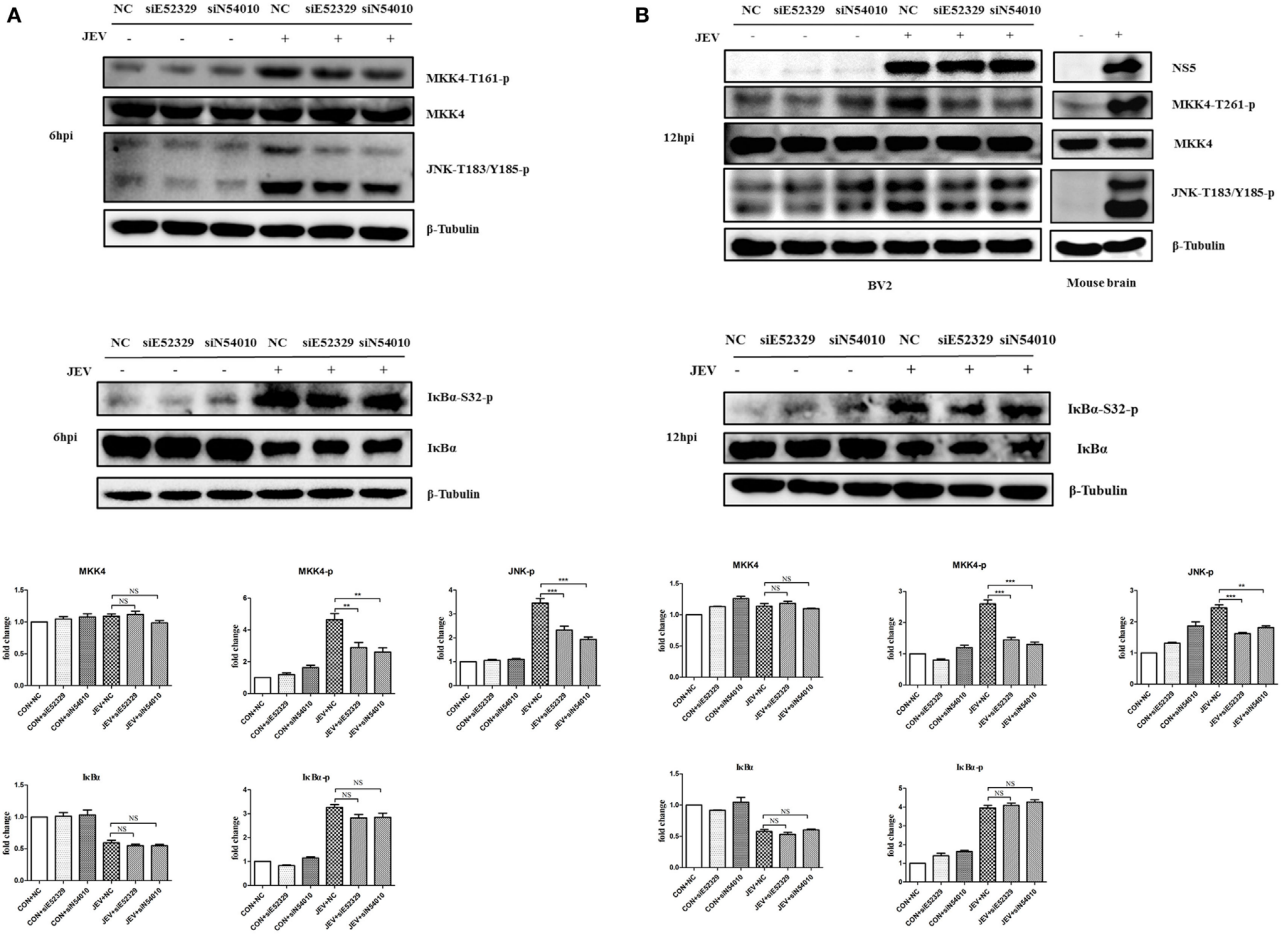
Selected long non-coding RNAs (lncRNAs) regulate MKK4 phosphorylation and mediate Japanese encephalitis virus (JEV)-induced inflammatory response. Immunoblot analysis of MKK4, JNK, and IκBα phosphorylation in JEV-infected cells and mice brain. BV2 cells were transfected with siE52329, siN54010, or their non-specific control small-interfering RNA (siRNA, final concentration, 50 nM) for 24 h, and then infected with JEV at multiplicity of infection (MOI) of 5 for 6 h **(A)** or 12 h **(B)**. BALB/c mice were inoculated intracranially with 20 µl of phosphate-buffered saline (PBS) or 50 plaque-forming units (PFU) of JEV in 20 µl of PBS, and the brain tissues were collected at 5 days postinfection. The extracts from BV2 cells and mouse brain homogenate were subjected to Western blot analysis with antibodies recognizing the indicated proteins. Levels of phosphorylated MKK4, IκBα, and JNK of BV2 cells were quantified by ImageJ software (NIH, Bethesda, MD, USA) and normalized to the amount of β-tubulin. After the quantification, one-way ANOVA analysis was performed (*P < 0.05, **P < 0.01, ***P < 0.001). Data are representative of three independent experiments.

Our previous study demonstrated that JNK plays an important role in JEV-mediated inflammation (12), and we found that MKK4/7, the upstream kinase of JNK, is the most enriched pathway in this study. Therefore, we wondered whether the selected lncRNAs (E52329 and N54010) can regulate the MKK/JNK pathway. To validate it, we first examined the phosphorylation of MKK4 at Thr^261^ and JNK at Thr^183^ and Tyr^185^ in JEV-infected mouse brain samples (Figure 7B). Each of these phosphorylation events is associated with increased kinase activity (40, 41). To examine the role of lncRNA E52329 and N54010 in regulating the kinase activity of MKK4/JNK pathway, BV2 cells were transfected with siE52329, siN54010 or non-specific control siRNA, and then infected with JEV. Our findings showed that reduction of these lncRNAs significantly decreased the kinase activity of MKK4 and JNK after JEV infection at 6 and 12 h (Figures 7A,B). On the other hand, NFκB is known as one of the key transcriptional factors of inflammatory cytokines in addition to AP-1 which can be activated by JNK. We also measured whether the lncRNA E52329 and N54010 can modify the NFκB pathway. We found that the phosphorylation of IκBα, a direct inhibitor of NFκB, was increased and IκBα protein was degraded upon JEV infection, but blocking the lncRNAs did not alter the phosphorylation level and protein amount of IκBα (Figures 7A,B). Thus, we consider that the selected lncRNAs may regulate JEV-induced inflammation through activation of MKK4/JNK pathway but not NFκB pathway.

## Discussion

The struggles between viruses and hosts have been existed for very long times. People usually gave attention to the interaction between protein-coding genes and viruses. Interestingly, only 2% of the mammalian genome is translated into proteins, and the remaining genome contains the non-coding RNA genes (42). Several transcriptome profiling studies have provided compelling evidence highlighting the importance of non-coding RNAs, such as miRNAs, in modulating the immune response against various viral infections, including JEV infection (43). However, the roles of lncRNAs in viral infections and associated inflammatory conditions are largely unknown.

Long non-coding RNAs have emerged as important regulators of gene expression, with an accumulating body of evidence linking lncRNAs to pathologies, including inflammatory diseases (44). Albeit, the precise functions of lncRNAs in virus-mediated pathogenesis remain poorly understood, evidence from recent studies indicates that lncRNAs may play a key role in virus-mediated inflammatory responses (19, 20). Herein, a transcriptomic profiling of lncRNAs and protein-coding genes from JEV-infected mice brain was performed using a microarray platform. The results of our study reveal the first experimental evidence demonstrating the complex regulation of lncRNAs by JEV infection in mice brain and microglial cells. The present study is unique from different angles. First, we used mice brain to understand the regulation of host lncRNAs upon JEV infection. This may provide us a way to elucidate the relationship between lncRNAs and protein-coding genes in the brain, having the more complex biological system compare to cell culture system. Second, we validated our findings using microglial cells, which play important role in eliciting innate immune response during JEV infection (4). However, some of the lncRNAs were not detected in BV2 cells, which may associate with cell type specificity. Third, the integration of microarray platform, quantitative real-time PCR, GO analysis, pathways analysis, and lncRNA-mRNA coexpression network analysis has allowed us to conduct an active comparative genomics and bioinformatics study to reveal host lncRNAs expression patterns associated with JEV infection. We have identified a unique series of host molecular responses involving different combinatorial contributions of multiple cellular lncRNAs. This provides an opportunity to understand the unique cellular lncRNAs-mRNA interactome network, dynamically regulated by JEV infection. Since we confirmed our findings using microglia cell line, further validations using primary microglia remain to be done in future.

Several lncRNAs have been investigated to play important roles in modulating the host immune response during viral infections (45). For instance, lncRNA-CMPK2 and -NRAV have been found to negatively regulate interferon response (19, 46), which is an important component of innate immune system against viral infections (47). JEV non-structural protein NS5 can block interferon-α signaling as an immune evasion strategy (48). For this reason, we believe that lncRNAs may contribute to regulation of JEV-induced pathogenesis. To the best of our knowledge, this is the first report where complete lncRNAs and mRNAs are profiled in JEV-infected mice brain using microarray approach, and differentially regulated lncRNAs and mRNA transcripts are reported.

In order to understand the impact of global lncRNAs modulation during JEV infection, we further analyzed GO terms and cellular pathways that may have significance in JEV pathogenesis. We found that the most significant GOs associated with upregulated transcripts were related to innate immune response, inflammatory response, apoptotic process, acetylation, nucleotide binding, and defense response to virus infections; whereas the GOs terms associated with downregulated transcripts were mainly limited to ions transportation, cell adhesion, signal transduction, and synaptic transmission. Similarly, the most enriched upregulated pathways included Jak-STAT signaling pathway, Toll-like receptor signaling pathway, MAPK signaling pathways, and pathways related to Herpes simplex and influenza A viruses infection. In contrast, the most enriched downregulated pathways were related to calcium signaling, neuroactive ligand–receptor signaling, and retrograde endocannabinoid signaling. To find the potential key lncRNAs involved during JEV infection, we built the lncRNA-mRNA and lncRNA-lncRNAs coexpression networks. To verify the coexpression Network, we performed RNA pull-down experiments and found that three mRNAs Nod1, Tap2, and Col4a1can interact with indicated lncRNAs. Nod1 are known to play a remarkable role in host immune responses which can positively regulate JNK cascade, NFκB activity (49). This may explain the fact of reduced production of inflammatory cytokines upon silencing of target genes. Our analysis revealed some important genes that may have their roles in regulating JEV-induced neuroinflammation. Considering the networks analyses and previous study, we speculated that the selected genes may trigger the MKK4/JNK and NFκB pathway, and our experimental found the selected lncRNAs may activate MKK4/JNK pathway but not NFκB pathway. The lncRNA E52329 and N54010 may also regulate other pathways to alter the JEV-mediated inflammation. However, we can not exclude all the other possibilities by experiments. We also compared our mRNA transcriptomic profile with a recent study revealing the dynamic changes in miRNAome and transcriptome in JEV-infected microglia (50). Of the 30 enlisted differentially expressed genes, 10 genes are also found to be differentially expressed in our study. Moreover, nine of 11 pathways predicted in referenced study are also analogous to our prediction analysis, thus, supporting our predictions.

In conclusion, we first generated the expression profile of lncRNAs and related mRNAs in JEV-infected mice brain based on a microarray approach. Using the bioinformatics tools, we found some important lncRNAs that may involve during JEV infection. Furthermore, our experimental data revealed the role of selected lncRNAs in regulating the kinase activity of MKK4 and JNK, which are considered to be involved in inflammatory pathway, and thus, confirmed our predictions. This study may provide insights into the molecular mechanisms involved in JEV pathogenesis. Further studies are still required to understand the biological functions of these identified lncRNAs during JEV infection. As the roles of lncRNAs in viral infections have not yet been fully identified and understood, this study may also provide valuable resource for further studies.

## Ethics Statement

Adult BALB/C mice (6 weeks old) were purchased from Hubei Provincial Center for Disease Control and Prevention, Wuhan, China. Mice were randomly assigned to two groups (*n* = 3 for each group): the JEV-infected group and the control group. For the infection group, mice were inoculated intracranially with 200 PFU JEV (P3 strain) in 20 μl DMEM, whereas mice belonging to the control group were injected intracranially with equal volume of DMEM. On day 5 postinfection, mice infected with JEV showed signs of acute encephalitis. Mice from both groups were euthanized, and brain samples were collected for further studies. All animal experiments were performed following the National Institute of Health Guide for the Care and Use of Laboratory Animals, and the experimental protocols were approved by the Research Ethics Committee of College of Veterinary Medicine, Huazhong Agricultural University, Hubei, Wuhan, China (No. S02914040M).

## Author Contributions

YL, HZ, and BZ conceived the experiments. YL, HZ, and ZC performed the experiments. DZ, BZ, and BHZ conceived the animal experiments. UA, JY, QX, and SC analyzed and interpreted the data. HZ, UA, JY, and QX wrote the original draft of the manuscript. YS, HC, and SC provided critical comments. JY, YS, and SC approved the final version of manuscript. HC and SC secured funding.

## Conflict of Interest Statement

The authors declare that the research was conducted in the absence of any commercial or financial relationships that could be construed as a potential conflict of interest.

## Supplementary Material

Figure S1 Work flow of the microarray analysis.

Figure S2 Top ten gene annotation and KEGG pathway annotation of differentially expressed genes. **(A,B)** The functional annotation of upregulated **(A)** and downregulated **(B)** mRNAs. **(C,D)** The functional annotation of upregulated **(C)** and downregulated **(D)** lncRNAs. Red color indicates upregulated genes, whereas blue color indicates downregulated genes. The intensity of color expresses the log *P*-value.

Figure S3 Coexpression network of lncRNA and mRNA. **(A)** lncRNA-mRNA coexpression network in mock-infected group. **(B)** lncRNA-mRNA coexpression network in JEV-infected group.

Figure S4 Selected lncRNAs regulate JEV-induced production of inflammatory cytokines. **(A)** RAW cells were transfected with siE52329, siN54010, or their non-specific control siRNA (final concentration, 50 nM) for 24 h, and then expression levels of lncRNA NONMMUT054010 and ENSMUST00000152329 were detected by quantitative real-time PCR. **(B,C)** RAW cells were transfected with siE52329, siN54010, or their non-specific control siRNA (final concentration, 50 nM) for 24 h, and then infected with JEV at MOI of 5 for 2 h. The mRNA **(B)** and protein **(C)** levels of TNF-α, IL-6, and IL-1β were analyzed by quantitative real-time PCR and ELISA, respectively. IFN-β mRNA level was determined by quantitative real-time PCR. **P* < 0.05; ***P* < 0.01. **(D)** RAW cells were transfected with siE52329, siN54010, or their non-specific control siRNA (final concentration, 50 nM) for 24 h, and then infected with JEV at MOI of 5 for the indicated times. The titers of infectious virus in the culture supernatants were determined by plaque assay. One-way ANOVA with subsequent Bonferroni’s Multiple Comparison. All data are representative of three independent experiments.

Table S1 The annotation of lncRNAs and mRNAs in the database of Refseq, Ensembl, and non-code.

Table S2 Differentially expressed lncRNAs and mRNAs between JEV-infected and mock-infected groups.

Tables S3 and S4 Go terms and pathways analyses of differentially expressed lncRNAs and mRNAs.

Table S5 lncRNA-mRNA coexpression network in each of the two separate groups.

Table S6 The sequence of primers and siRNAs.
