# Supplementary figures and images for: Microarray Analysis Identifies the Potential Role of Long Non-Coding RNA in Regulating Neuroinflammation during Japanese Encephalitis Virus Infection
Source: Front Immunol. 2017 Sep 29;8:1237. doi: 10.3389/fimmu.2017.01237 (PMC5626832; doi:10.3389/fimmu.2017.01237)

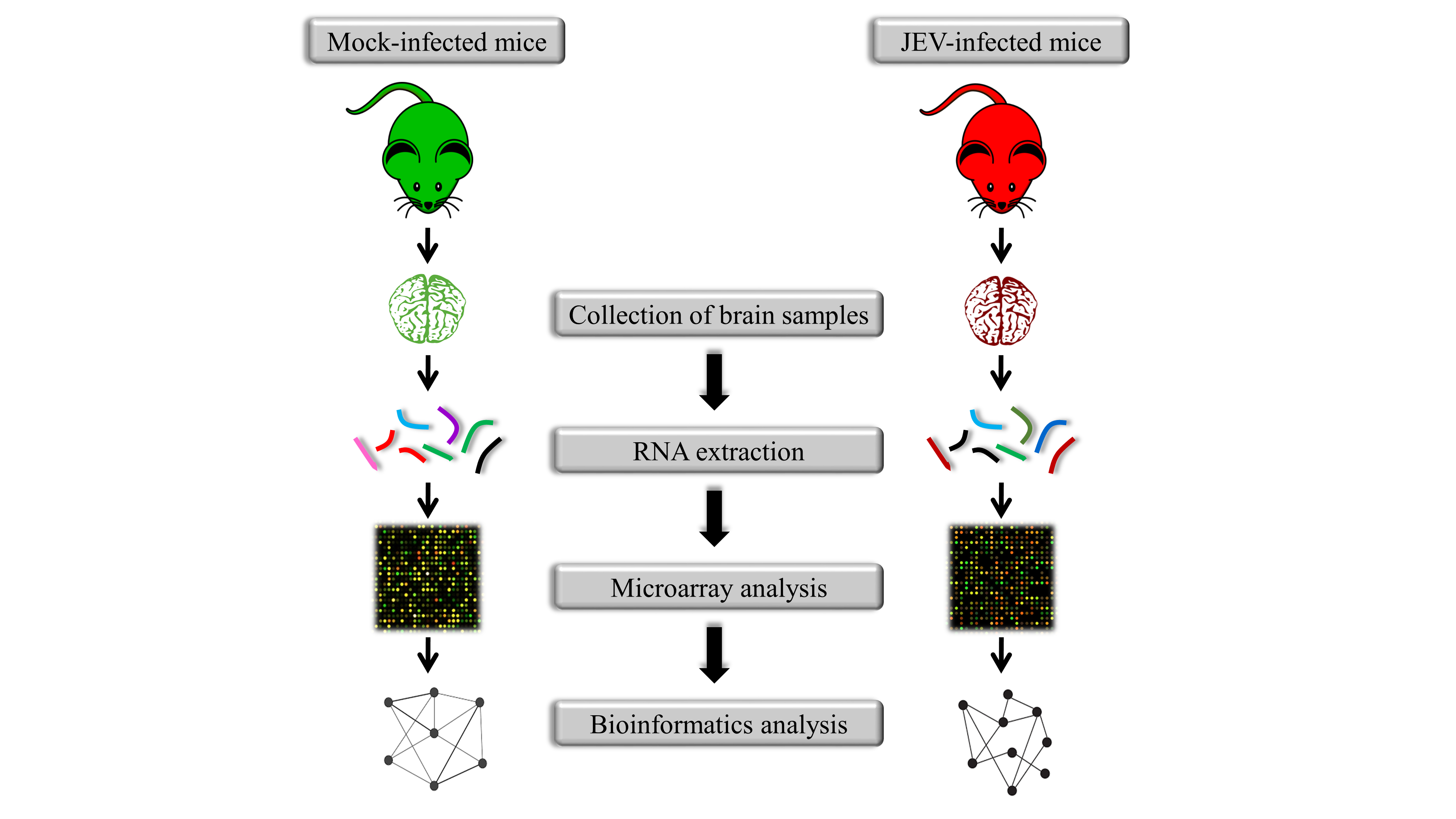

Supplement: Figure S1 — Work flow of the microarray analysis. [file image_1.tif]

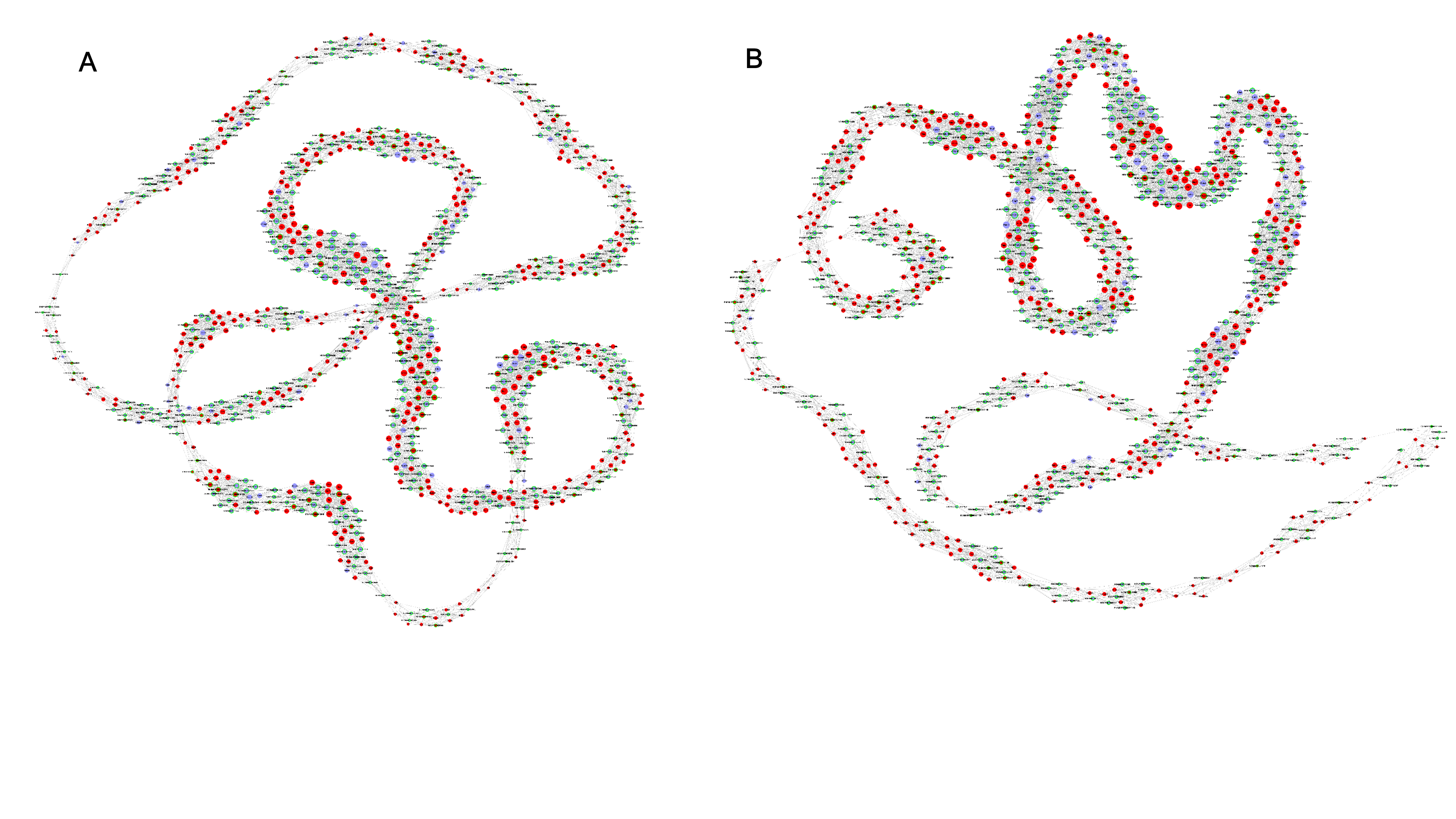

Supplement: Figure S3 — Coexpression network of lncRNA and mRNA. (A) lncRNA-mRNA coexpression network in mock-infected group. (B) lncRNA-mRNA coexpression network in JEV-infected group. [file image_3.tif]

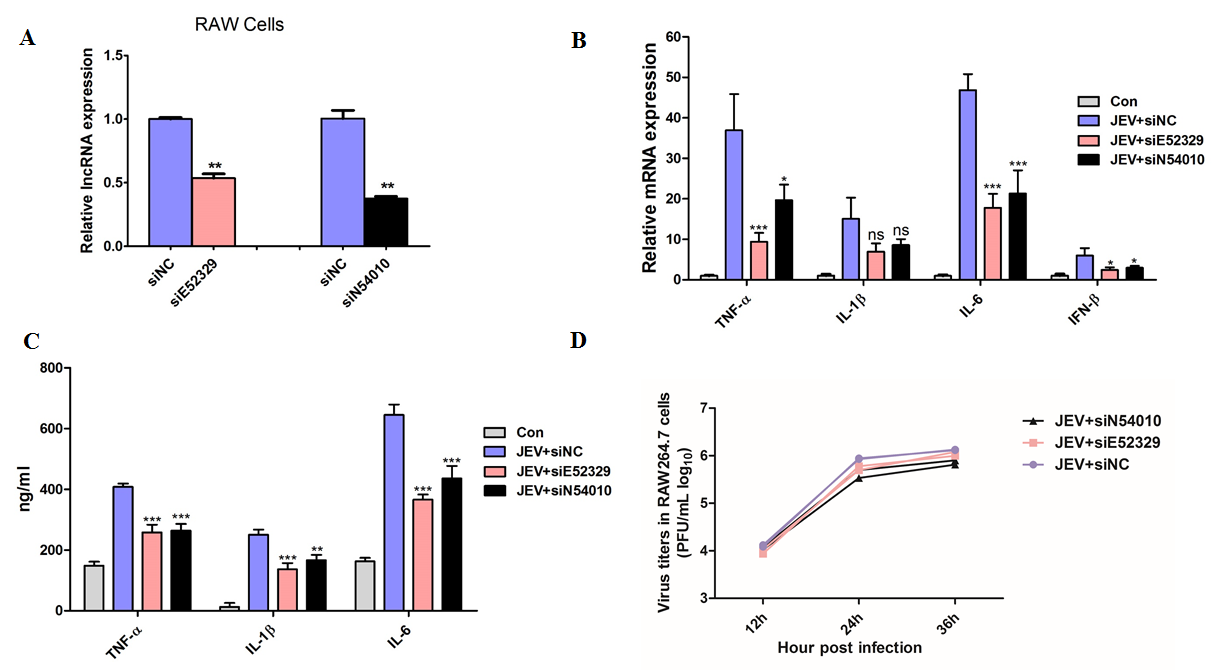

Supplement: Figure S4 — Selected lncRNAs regulate JEV-induced production of inflammatory cytokines. (A) RAW cells were transfected with siE52329, siN54010, or their non-specific control siRNA (final concentration, 50 nM) for 24 h, and then expression levels of lncRNA NONMMUT054010 and ENSMUST00000152329 were detected by quantitative real-time PCR. (B,C) RAW cells were transfected with siE52329, siN54010, or their non-specific control siRNA (final concentration, 50 nM) for 24 h, and then infected with JEV at MOI of 5 for 2 h. The mRNA (B) and protein (C) levels of TNF-α, IL-6, and IL-1β were analyzed by quantitative real-time PCR and ELISA, respectively. IFN-β mRNA level was determined by quantitative real-time PCR. *P < 0.05; **P < 0.01. (D) RAW cells were transfected with siE52329, siN54010, or their non-specific control siRNA (final concentration, 50 nM) for 24 h, and then infected with JEV at MOI of 5 for the indicated times. The titers of infectious virus in the culture supernatants were determined by plaque assay. One-way ANOVA with subsequent Bonferroni’s Multiple Comparison. All data are representative of three independent experiments. [file image_4.tif]
